# Development and Validation of a Sensitive and Robust Multiplex Antigen Capture Assay to Quantify Streptococcus pneumoniae Serotype-Specific Capsular Polysaccharides in Urine

**DOI:** 10.1128/msphere.00114-22

**Published:** 2022-08-01

**Authors:** Gowrisankar Rajam, Yuhua Zhang, Joseph M. Antonello, Rebecca J. Grant-Klein, Lauren Cook, Reshma Panemangalore, Huy Pham, Stephanie Cooper, Thomas D. Steinmetz, Jennifer Nguyen, Mathias W. Pletz, Grit Barten-Neiner, Rocio D. Murphy, Leonard J. Rubinstein, Katrina M. Nolan

**Affiliations:** a Merck & Co., Inc., Rahway, New Jersey, USA; b Institute for Infectious Diseases and Infection Control, Jena University Hospital, Jena, Thuringia, Germany; c CAPNETZ Stiftung, Hannover, Germany; d Biomedical Research in Endstage and Obstructive Lung Disease Hannover, Hannover, Germany; Duke Human Vaccine Institute

**Keywords:** Luminex, pneumococcal disease, community-acquired pneumonia, multiplex assay, pneumococcal vaccine, urine, vaccine serotypes

## Abstract

Streptococcus pneumoniae is a major cause of community-acquired pneumonia (CAP) in young children, older adults, and those with immunocompromised status. Since the introduction of pneumococcal vaccines, the burden of invasive pneumococcal disease caused by vaccine serotypes (STs) has decreased; however, the effect on the burden of CAP is unclear, potentially due to the lack of testing for pneumococcal STs. We describe the development, qualification, and clinical validation of a high-throughput and multiplex ST-specific urine antigen detection (SSUAD) assay to address the unmet need in CAP pneumococcal epidemiology. The SSUAD assay is sensitive and specific to the 15 STs in the licensed pneumococcal conjugate vaccine V114 (STs 1, 3, 4, 5, 6A, 6B, 7F, 9V, 14, 18C, 19A, 19F, 22F, 23F, and 33F) and uses ST-specific monoclonal antibodies for rapid and simultaneous quantification of the 15 STs using a Luminex microfluidics system. The SSUAD assay was optimized and qualified using healthy adult urine spiked with pneumococcal polysaccharides and validated using culture-positive clinical urine samples (*n* = 34). Key parameters measured were accuracy, precision, sensitivity, specificity, selectivity, and parallelism. The SSUAD assay met all prespecified validation acceptance criteria and is suitable for assessments of disease burden associated with the 15 pneumococcal STs included in V114.

**IMPORTANCE** Streptococcus pneumoniae has more than 90 serotypes capable of causing a range of disease manifestations, including otitis media, pneumonia, and invasive diseases, such as bacteremia or meningitis. Only a minority (<10%) of pneumococcal diseases are bacteremic with known serotype distribution. Culture and serotyping of respiratory specimens are neither routine nor reliable. Hence, the serotype-specific disease burden of the remaining (>90%) noninvasive conditions is largely unknown without reliable laboratory techniques. To address this need, a 15-plex urine antigen detection assay was developed and validated to quantify pneumococcal serotype-specific capsular polysaccharides in urine. This assay will support surveillance to estimate the pneumococcal disease burden and serotype distribution in nonbacteremic conditions. Data obtained from this assay will be critical for understanding the impact of pneumococcal vaccines on noninvasive pneumococcal diseases and to inform the choice of pneumococcal serotypes for next-generation vaccines.

## INTRODUCTION

Despite vaccine availability, Streptococcus pneumoniae continues to be a major respiratory pathogen in children and older adults (1, 2). Pneumococci exhibit capsular polysaccharide diversity, drug resistance, and the ability to cause a spectrum of diseases, from otitis media to potentially life-threatening meningitis, contributing to their role as a major public health burden (1–7). Structural differences in the capsule polysaccharide, a key virulence factor of S. pneumoniae, are used to categorize pneumococcal serotypes (STs), with more than 90 distinct STs identified to date (2, 8). Pneumococcal pneumonia can be invasive (bacteremic) or noninvasive (nonbacteremic), with the latter being responsible for the majority of pneumococcal disease. The incidence of nonbacteremic pneumonia is difficult to estimate, primarily due to the limited availability of *in vitro* assays (9).

A pneumococcal polysaccharide (PnPs) vaccine covering 23 STs (PPSV23), as well as pneumococcal conjugate vaccines (PCVs) covering 10 or 13 STs (PCV10 and PCV13), are currently available (8, 10). PCV13 is currently used in the childhood immunization schedule, while PPSV23 is recommended for older adults and children >2 years of age with high-risk conditions in many European countries and the United States (11–14). A 20-valent PCV (PCV20) and an adjuvanted 15-valent PCV (V114; Vaxneuvance; Merck Sharp & Dohme LLC, a subsidiary of Merck & Co., Inc., Rahway, NJ, USA) have recently been approved in the United States by the Food and Drug Administration (FDA) for adults ≥18 years of age; both offer broader ST coverage than PCV13 (15, 16).

Community-acquired pneumonia (CAP) is one of the most common infectious diseases in children and adults (17–21). Sensitive and specific assays are essential to determine the burden of pneumococcal disease (PD) in conditions such as CAP. Currently, pneumococcal CAP is diagnosed with nonspecific tests, such as chest X-rays and empirical clinical symptoms (22, 23). Occasionally, BinaxNOW, a urine antigen test that detects pneumococcal cell wall polysaccharide, is used for CAP diagnosis; however, this assay is not ST specific (24). Routine ST identification in patients with invasive pneumococcal disease (IPD) is done via microbiological culture of clinical samples followed by a highly subjective latex agglutination and/or Quellung test, both of which have low sensitivity and require viable bacterial samples (25–28). Highly accurate ST identification can be achieved with polymerase chain reaction (PCR) and whole-genome analysis, but these are not suitable for rapid or repeated testing to support large-scale surveillance studies or clinical trials (29). More recently, monoclonal antibodies have been used to detect and identify the presence of ST-specific PnPs in clinical samples (25, 30, 31).

Here, we describe the development, qualification, and clinical validation of a high-throughput, multiplex, serotype-specific urine antigen detection (SSUAD) assay for the quantification of PnPs of S. pneumoniae (STs 1, 3, 4, 5, 6A, 6B, 7F, 9V, 14, 18C, 19A, 19F, 22F, 23F, and 33F) covered by V114, a recently licensed 15-valent PCV. We report performance characteristics that establish SSUAD as a suitable method to detect ST-specific PnPs in adult urine to support epidemiology studies or clinical trials.

## RESULTS

### Assay development and optimization.

**FIG 1:**
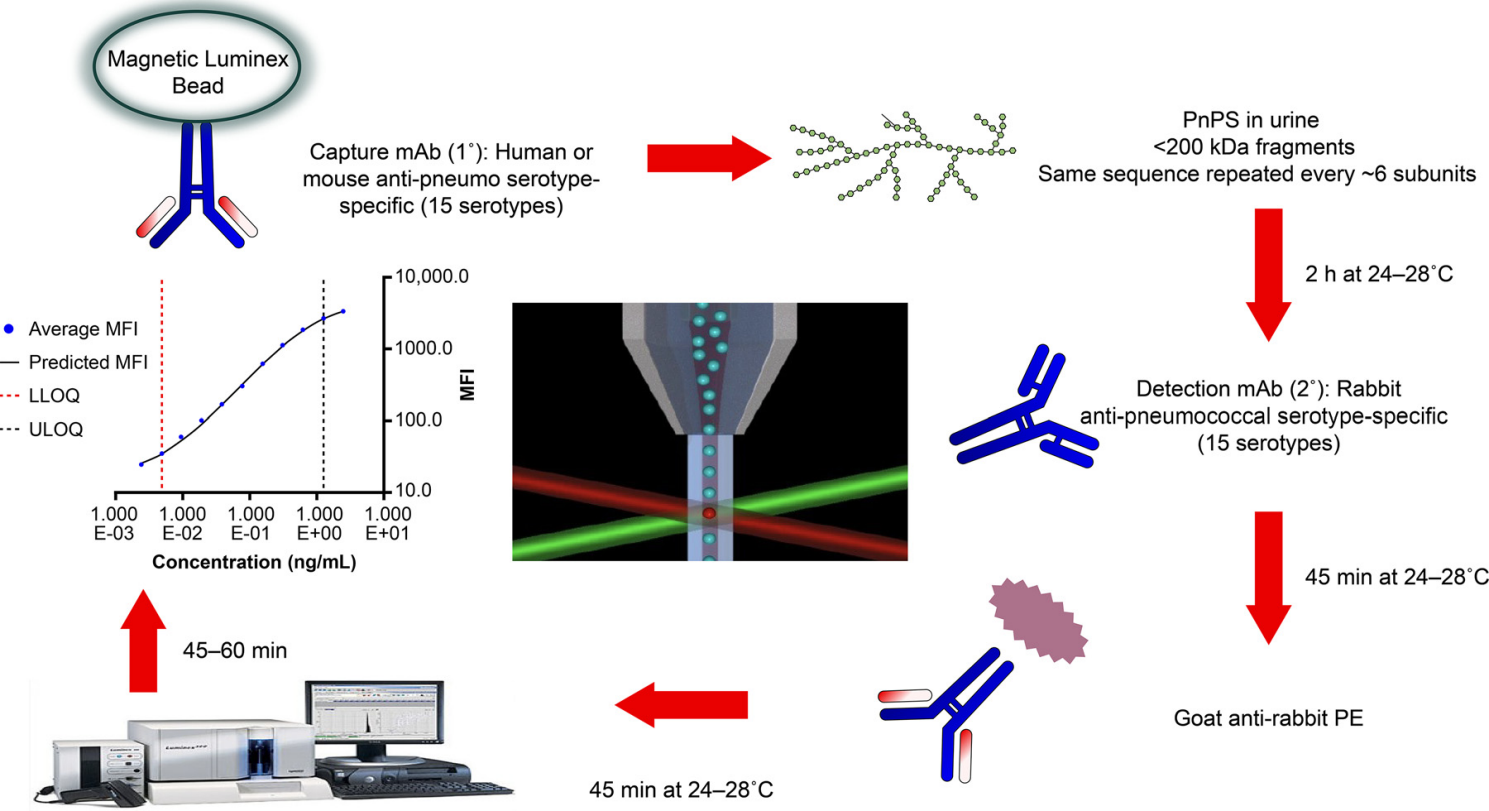
Pneumococcal urine antigen detection assay principle and workflow. LLOQ, lower limit of quantitation; mAb, monoclonal antibody; MFI, median fluorescence intensity; PE, phycoerythrin; PnPs, pneumococcal polysaccharide; ULOQ, upper limit of quantitation.

The SSUAD assay was developed and optimized to detect and quantify V114 serotypes in 15-plex format (Fig. 1). During the multiplex optimization process, STs 1, 7F, 18C, 19F, and 23F required additional adjustments to their capture monoclonal antibody (mAb) and secondary mAb reaction concentrations. The final optimized SSUAD assay conditions are provided in Table 1, and quality control (QC) acceptance limits are provided in Table S1 in the supplemental material.

**TABLE 1:** Multiplex SSUAD-optimized reagent concentrations and assay conditions^*a*^

Pn serotype	Capture mAb coating concentration (μg/mL)	sAb concentration at the working dilution (μg/mL)	In-well concentration (ng/mL)^*b*^				
			QC1^*a*^	QC2^*a*^	QC3^*a*^	QC4^*a*^	Reference standard
1	1.185	0.09	0.125	0.031	0.008	0.00	0.50
3	0.25	0.05	0.125	0.031	0.008	0.00	0.50
4	0.25	0.18	0.500	0.125	0.031	0.00	2.00
5	0.25	1.67	0.500	0.125	0.031	0.00	2.00
6A	1.053	1.52	0.625	0.156	0.039	0.00	2.50
6B	0.50	9.58	0.125	0.031	0.008	0.00	0.50
7F	1.00	1.55	2.500	0.625	0.156	0.00	10.00
9V	1.00	1.03	0.250	0.063	0.016	0.00	1.00
14	0.25	0.84	0.500	0.125	0.031	0.00	2.00
18C	0.25	9.97	0.500	0.125	0.031	0.00	2.00
19A	0.534	0.09	0.125	0.031	0.008	0.00	0.50
19F	0.134	0.01	2.500	0.625	0.156	0.00	10.00
22F	0.833	0.68	0.500	0.125	0.031	0.00	2.00
23F	0.533	0.01	5.000	1.250	0.313	0.00	20.00
33F	0.133	0.20	0.500	0.125	0.031	0.00	2.00

a Tertiary antibody working dilution was 1:500. Primary incubation was 2 h ± 10 min at 23 ± 2°C with 600- to 800-rpm agitation. Secondary incubation was 45 min ± 5 min at 23 ± 2°C with 600- to 800-rpm agitation. Tertiary incubation was 45 min ± 5 min at 23 ± 2°C with 600- to 800-rpm agitation. mAb, monoclonal antibody; Pn, pneumococcal; QC, quality control; sAb, secondary mAb; SSUAD, serotype-specific urine antigen detection.

b QC acceptance limits are provided in Table S1.

10.1128/msphere.00114-22.1 TABLE S1 Control sample acceptance limits (in nanograms per milliliter) by control sample and serotype. LLOQ, lower limit of quantitation; ULOQ, upper limit of quantitation. Download Table S1, DOCX file, 0.02 MB. Copyright © 2022 Rajam et al. 2022 Rajam et al. https://creativecommons.org/licenses/by/4.0/ This content is distributed under the terms of the Creative Commons Attribution 4.0 International license.

### Assay qualification. 

**FIG 2:**
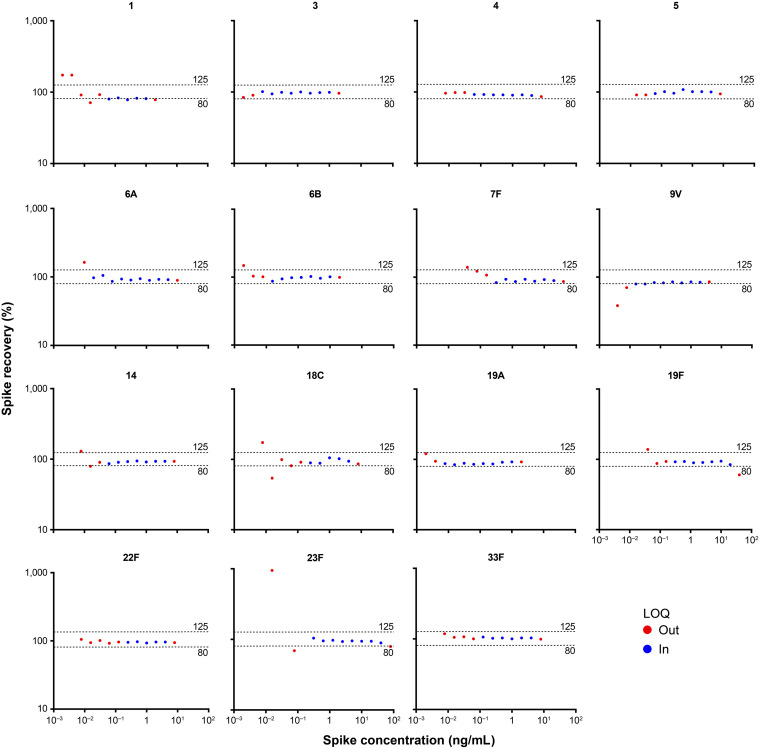
Relative accuracy (qualification). Horizontal dashed lines indicate 80% and 125% ranges. Results for sample 8 from serotype 1 were excluded. LOQ, limit of quantitation.

**(i) Accuracy.** The average recovery of 15 V114 PnPs in spiked samples was within the expected range of 80 to 125% throughout the defined quantifiable range for 13 of the 15 STs (Fig. 2). Throughout the defined quantifiable range, ST 9V had two spike concentrations (0.0156 and 0.0325 ng/mL), of which average recoveries were both 79%; the average recovery of ST 1 was outside the expected range for three spike concentrations (76 to 79%), primarily due to one sample that had very low relative accuracy estimates, weighing down the average recovery. Excluding that sample, the average recovery of ST 1 was within the expected range of 80 to 125%, except for 78% recovery at the 0.25-ng/mL spike concentration.

**(ii) Precision.** Across all V114 STs, intra-assay precision estimates ranged between 4.9% and 8.5%, while total precision estimates ranged between 7.1% and 14.2% (Table 2). Across the 15 V114 STs, all differences between the analysts were within ±15% and all pairwise differences between Luminex readers were within ±10% (Table 2).

**TABLE 2:** Consolidated table of parameters from SSUAD qualification experiments

Assay characteristic	Value for serotype:														
	1	3	4	5	6A	6B	7F	9V	14	18C	19A	19F	22F	23F	33F
LLOQ 1:4 dil. corrected (ng/mL)	0.0625	0.0078	0.0313	0.0313	0.0781	0.0156	0.3125	0.0156	0.0313	0.25	0.0078	1.0	0.25	0.3125	0.125
ULOQ 1:4 dil. corrected (ng/mL)	1	1	4	4	5	1	20	2	4	4	1	20	4	40	4
Total precision (CV [%])	11.8	8.7	8.9	8.5	9.2	10.1	10.4	8.9	7.1	14.2	8.3	9.6	10.8	13.1	13.7
Assay precision to analyst (mean % difference in concentration)	10.2	5.8	6.5	6.2	5.8	10.0	7.7	4.9	4.9	11.0	5.8	6.7	8.8	11.7	7.9
Assay precision to instrument (mean % difference in concentration)	5.6	3.3	3.0	3.4	2.2	3.2	4.6	2.5	3.3	5.8	4.7	5.1	2.7	7.3	8.4
On-target specificity (% recovery across absent serotypes)	65	51	89	79	75	97	101	73	78	58	83	91	73	78	76
Off- target specificity (PnPs concentration [ng/mL])	<LLOQ	<LLOQ	<LLOQ	<LLOQ	<LLOQ	<LLOQ	<LLOQ	<LLOQ	<LLOQ	<LLOQ	<LLOQ	<LLOQ	<LLOQ	<LLOQ	<LLOQ
Accuracy range (PnPs concentration range [ng/mL] with 80–125% recovery)	0.0313–0.125	0.002–2	0.0078–8	0.0156–8	0.0195–10	0.0039–2	0.0781–40	0.0625–4	0.0313–8	0.0313–8	0.002–2	0.0781–20	0.0078–8	0.3125–40	0.0078–8
Selectivity (% recovery in neat urine)	119	99	108	110	110	109	105	91	101	101	101	107	113	108	110
Parallelism (% dilution bias per 10-fold dilution)	0	0	−6	3	4	14	−1	−7	3	74	−3	3	0	1	7

CV, coefficient of variation; dil., dilution; LLOQ, lower limit of quantitation; PnPs, pneumococcal polysaccharide; SSUAD, serotype-specific urine antigen detection; ULOQ, upper limit of quantitation.

**(iii) Sensitivity and LOQ.** Dilution-corrected limits of quantitation (LOQ) determined in the qualification study are given in Table 2. Owing to the specificity testing results for ST 19F (see “Specificity,” below), ST 19F at 1.00 ng/mL had the highest lower limit of quantitation (LLOQ).

**(iv) Specificity.** For 13 of the 15 V114 STs, average measured concentrations for spiked PnPs were within 2-fold of spike concentrations; however, for ST 3 and ST 18C, the measured concentrations approached 50% of the nominal spike concentration (Table 3). For 14 of the 15 V114 STs, measured concentrations for the missing PnPs were below their determined LLOQ. The average measured concentration for ST 19F was 0.485 ng/mL for the ST 19F missing sample, indicating potential interference at the current LLOQ. Based on this observation, the dilution-corrected LLOQ for ST 19F was set to 1.00 ng/mL.

**TABLE 3:** Assay specificity (qualification)—geometric mean PnPs concentration ratios by serotype and missed serotype^*a*^

Missing type	Spike recovery ratio (measured concentration/spike concentration) for serotype:														
	1	3	4	5	6A	6B	7F	9V	14	18C	19A	19F	22F	23F	33F
1	<LLOQ	0.53	1.02	0.76	0.75	1.01	1.02	0.71	0.87	0.61	0.80	0.88	0.77	0.73	0.79
3	0.62	<LLOQ	1.01	0.77	0.74	0.99	0.98	0.71	0.80	0.58	0.79	0.87	0.83	0.69	0.77
4	0.62	0.49	<LLOQ	0.82	0.74	1.02	1.01	0.71	0.82	0.57	0.78	0.87	0.80	0.73	0.77
5	0.66	0.44	1.05	<LLOQ	0.73	0.95	1.05	0.75	0.83	0.54	0.85	0.98	0.82	0.84	0.82
6A	0.67	0.42	0.97	0.80	<LLOQ	1.02	1.06	0.73	0.83	0.54	0.82	0.94	0.81	0.79	0.83
6B	0.70	0.48	1.14	0.79	0.71	<LLOQ	1.04	0.72	0.80	0.55	0.82	0.91	0.80	0.74	0.81
7F	0.60	0.59	0.86	0.75	0.68	1.04	<LLOQ	0.71	0.74	0.57	0.81	0.86	0.74	0.69	0.80
9V	0.63	0.57	0.89	0.77	0.69	1.01	1.02	<LLOQ	0.73	0.63	0.82	0.88	0.77	0.75	0.79
14	0.61	0.59	0.89	0.72	0.69	0.95	1.01	0.72	<LLOQ	0.53	0.81	0.86	0.76	0.73	0.81
18C	0.60	0.53	0.92	0.81	0.67	0.93	1.04	0.73	0.74	<LLOQ	0.85	0.95	0.80	0.77	0.79
19A	0.73	0.54	0.86	0.78	0.70	0.98	1.05	0.76	0.78	0.59	<LLOQ	0.81	0.78	0.78	0.80
19F	0.67	0.53	0.84	0.78	0.68	1.01	1.01	0.72	0.74	0.51	0.82	0.10	0.80	0.75	0.75
22F	0.66	0.52	0.85	0.74	0.68	1.03	1.02	0.71	0.73	0.55	0.82	0.92	<LLOQ	0.74	0.77
23F	0.65	0.55	0.88	0.79	0.69	0.98	1.03	0.73	0.79	0.58	0.83	0.96	0.81	<LLOQ	0.83
33F	0.63	0.50	0.81	0.74	0.66	0.90	0.98	0.69	0.70	0.49	0.81	0.90	0.76	0.71	<LLOQ
Across samples	0.70	0.42	0.89	0.81	0.71	0.91	1.07	0.74	0.82	0.46	0.85	1.02	0.86	0.86	0.88

a Concentration ratios of <0.5 are shaded. LLOQ, lower limit of quantitation; PnPs, pneumococcal polysaccharide.

**FIG 3:**
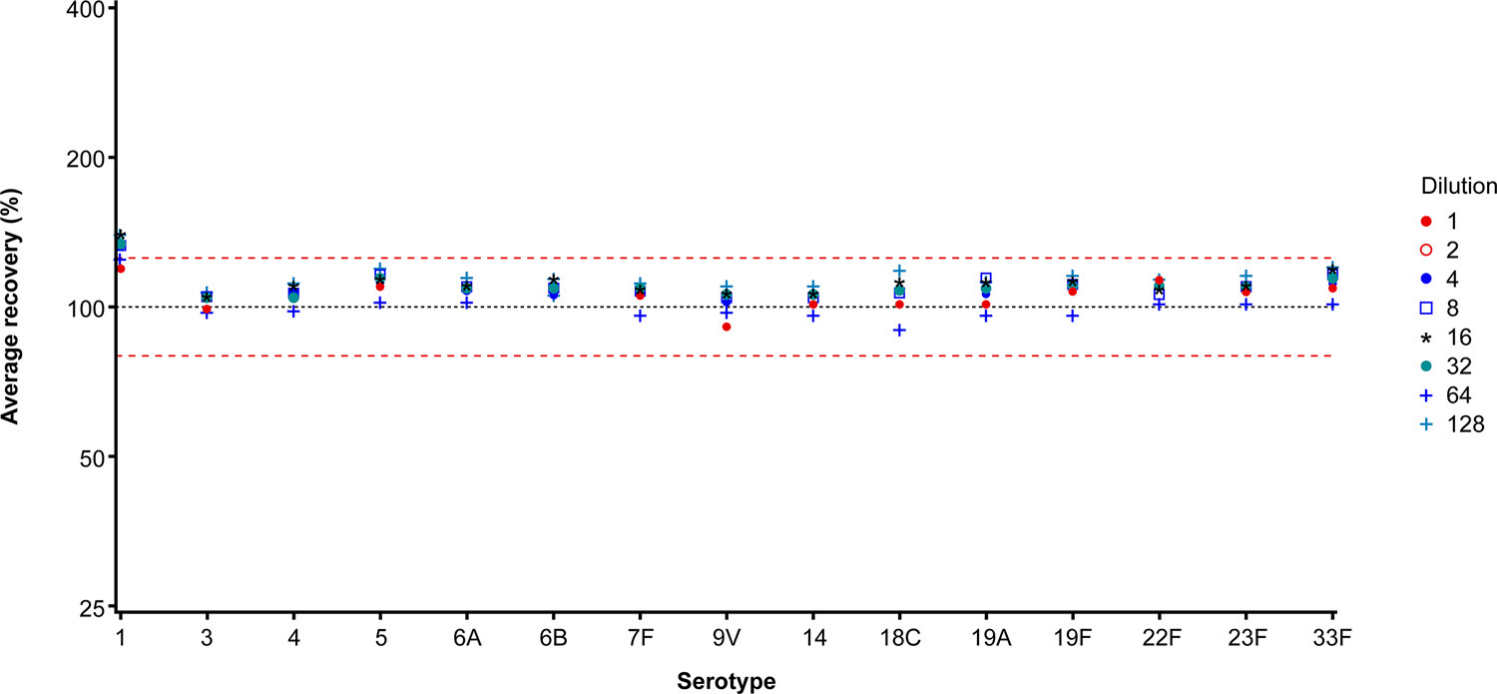
Assay selectivity (qualification); average recovery by ST and predilution of urine samples. Horizontal dashed lines show 80% and 125% ranges. ST, serotype.

**(v) Selectivity.** For 14 of the 15 V114 STs, the average recovery was within the expected range of 80 to 125% at each predilution level of urine (Fig. 3). For ST 1, the average recovery was consistent but clustered around the upper limit of acceptability, from 118.6% in neat urine to 138.9% in urine prediluted 1:128.

**(vi) Parallelism.** The overall dilution bias estimates per 10-fold dilution were between −15% and +15% for 14 of the 15 STs. For ST 18C, the estimated dilution bias was +74% per 10-fold dilution.

### Assay validation.

The SSUAD assay validation parameters are described in Table S2, and consolidated results for all validation experiments are presented in Table 4. A summary of control failures by serotype and control sample during validation is provided in Table S3.

**TABLE 4:** Consolidated table of parameters from SSUAD validation experiments^*a*^

	Serotype														
Assay characteristic	1	3	4	5	6A	6B	7F	9V	14	18C	19A	19F	22F	23F	33F
LLOQ 1:4 dil. corrected (ng/mL)	0.0625	0.0078	0.0313	0.0313	0.0781	0.0156	0.3125	0.0156	0.0313	0.25	0.0078	1.0	0.25	0.3125	0.125
ULOQ 1:4 dil. corrected (ng/mL)	1	1	4	4	5	1	20	2	4	4	1	20	4	40	4
Total precision (CV [%])	30.7 (22.0)	12.8	14.4	67.1 (8.7)	8.4	20.1	12.7	24.9	15.7	NE	6.2	7.2	9.4	3.2	16.8
Assay precision to analyst (mean % difference in concentration)	0 (6.78)	3.33	−1.14	10.39 (−4.52)	−2.28	29.06	−3.59	−0.63	1.08	NE	−2.12	−1.45	−13.46	4.32	−2.90
Assay precision to bead lot (mean % difference in concentration)	35.63 (24.75)	10.65	10.36	19.50 (7.18)	3.06	5.57	4.28	−22.11	19.97	NE	−2.58	4.39	−2.97	−7.19	10.70
Assay precision to instrument (mean % difference in concentration)	3.68 (−1.26)	5.80	−14.57	66.55 (0.23)	−4.20	−0.35	−12.94	1.06	2.09	NE	0.00	4.75	2.18	−5.16	5.50
On-target specificity (% recovery across absent serotypes)	74	57	79	77	72	98	106	85	82	67	81	95	84	75	79
Off-target specificity (PnPs concentration [ng/mL])	<LLOQ	<LLOQ	<LLOQ	<LLOQ	<LLOQ	<LLOQ	<LLOQ	<LLOQ	<LLOQ	<LLOQ	<LLOQ	<LLOQ	<LLOQ	<LLOQ	<LLOQ
Accuracy range (PnPs concentration range [ng/mL] with 80–125% recovery)	0.0625–2	0.002–2	0.0078–8	0.0156–8	0.0781–10	0.0039–2	0.3125–40	0.0039–4	0.0078–8	0.0625–4	1−2	0.1563–20	0.0625–8	0.1563–80	0.0156–8
Selectivity (% recovery in neat urine)	99	100	92	105	104	101	102	96	102	91	71	104	104	96	108
Parallelism (% dilution bias per 10-fold dilution)	97	−1	−4	−4	−3	−3	9	−2	3	28	−12	−6	2	0	0

a The precision estimates for types 1 and 5 were estimated with (without) one extreme concentration. A summary of control failures by serotype and control sample during validation is provided in Table S3. CV, coefficient of variation; dil., dilution; LLOQ, lower limit of quantitation; NE, not evaluable; PnPs, pneumococcal polysaccharide; SSUAD, serotype-specific urine antigen detection; ULOQ, upper limit of quantitation.

10.1128/msphere.00114-22.2 TABLE S2 SSUAD assay validation parameters. Validation parameters were established according to the regulatory guidance described in the ICH Guideline M10 on Bioanalytical Method Validation or the FDA Bioanalytical Method Validation Guidance for Industry. CV, coefficient of variation; FDA, US Food and Drug Administration; ICH, International Council for Harmonisation; LLOQ, lower limit of quantitation; LOD, limit of detection; PnPs, pneumococcal polysaccharide; ULOQ, upper limit of quantitation. Download Table S2, DOCX file, 0.02 MB. Copyright © 2022 Rajam et al. 2022 Rajam et al. https://creativecommons.org/licenses/by/4.0/ This content is distributed under the terms of the Creative Commons Attribution 4.0 International license.

10.1128/msphere.00114-22.3 TABLE S3 Summary of control failures by serotype and control sample during validation. CI, confidence interval. Download Table S3, DOCX file, 0.02 MB. Copyright © 2022 Rajam et al. 2022 Rajam et al. https://creativecommons.org/licenses/by/4.0/ This content is distributed under the terms of the Creative Commons Attribution 4.0 International license.

**(i) Accuracy.** During validation experiments, the concentrations of V114 PnPs recovered in test samples were within 80 to 125% of spiked concentrations throughout the defined quantifiable range for 14 of the 15 V114 STs. Recovery of ST 19A was lower than expected (from 42 to 81%). Despite the underestimation, the 19A assay was judged sufficiently accurate to quantitate ST 19A PnPs in patient urine given (i) the acceptable precision of the assay down to a measured concentration of 0.0078 ng/mL, (ii) the acceptable linearity of the 19A assay throughout the quantifiable range, and (iii) the fact that the magnitude of the inaccuracy was not excessive, being within 2-fold throughout the quantifiable range.

**(ii) Precision.** Twelve of the 14 evaluable V114 STs had a percent coefficient of variation (%CV) of <25%, with total assay precision estimates between 3.2% and 24.9%. ST 18C could not be evaluated for precision due to the unavailability of 18C-positive clinical samples. ST 1 and ST 5 had relatively high %CVs of 30.7% and 67.1%, respectively, driven by a single observation in each case. Upon exclusion of the observations of the outlier, the %CVs for ST 1 and ST 5 were 22.0% and 8.8%, respectively.

Across the 14 evaluable STs and after excluding outliers noted for ST 1 and ST 5, all differences between levels of assay precision factors (analyst, bead lot, and Luminex instrument) were <30%.

**(iii) Sensitivity and LOQ.** The LOQs defined in the qualification study were supported by the validation study and are the recommended LOQs for clinical testing.

**(iv) Specificity.** For all 15 V114 STs, measured concentrations for missing STs were below their determined LLOQ (Table 4). Spike recovery ratios, defined as the ratio of the measured concentration to the spike concentration, were determined for each measured value (Table 5). By this measure, the SSUAD assay was determined to have acceptable specificity.

**TABLE 5:** Assay specificity (validation)—geometric mean PnPs concentration ratios by serotype and missed serotype

Missing type	Spike recovery ratio (measured concentration/spike concentration) for serotype:														
	1	3	4	5	6A	6B	7F	9V	14	18C	19A	19F	22F	23F	33F
1	<LLOQ	0.52	0.89	0.75	0.75	0.97	1.04	0.82	0.90	0.68	0.79	0.93	0.80	0.72	0.78
3	0.69	<LLOQ	0.89	0.76	0.78	0.96	1.05	0.84	0.84	0.73	0.79	0.92	0.90	0.71	0.77
4	0.75	0.50	<LLOQ	0.83	0.78	0.99	1.11	0.86	0.86	0.76	0.80	0.96	0.88	0.76	0.78
5	0.72	0.48	0.85	<LLOQ	0.71	0.91	1.02	0.83	0.82	0.64	0.80	0.94	0.82	0.76	0.78
6A	0.80	0.47	0.82	0.80	<LLOQ	1.01	1.10	0.87	0.87	0.69	0.83	1.01	0.88	0.78	0.83
6B	0.78	0.46	0.82	0.77	0.71	<LLOQ	1.06	0.85	0.84	0.66	0.81	0.94	0.83	0.74	0.79
7F	0.69	0.64	0.77	0.74	0.70	0.97	<LLOQ	0.83	0.78	0.63	0.80	0.94	0.79	0.70	0.79
9V	0.70	0.60	0.77	0.74	0.71	0.94	1.08	<LLOQ	0.77	0.76	0.82	0.91	0.84	0.75	0.77
14	0.71	0.65	0.79	0.76	0.74	0.98	1.06	0.87	<LLOQ	0.76	0.82	0.94	0.82	0.78	0.81
18C	0.72	0.64	0.79	0.80	0.69	1.01	1.07	0.88	0.80	<LLOQ	0.84	0.98	0.86	0.76	0.77
19A	0.83	0.62	0.76	0.79	0.73	1.05	1.11	0.90	0.83	0.72	<LLOQ	0.99	0.85	0.82	0.80
19F	0.81	0.56	0.77	0.81	0.72	1.02	1.08	0.87	0.82	0.66	0.85	<LLOQ	0.88	0.79	0.80
22F	0.76	0.60	0.75	0.75	0.70	0.98	1.06	0.84	0.78	0.56	0.81	0.97	<LLOQ	0.75	0.77
23F	0.70	0.64	0.78	0.76	0.71	0.94	1.04	0.84	0.80	0.64	0.82	0.99	0.86	<LLOQ	0.78
33F	0.69	0.60	0.70	0.73	0.69	0.92	1.00	0.82	0.72	0.58	0.79	0.93	0.79	0.70	<LLOQ
Across samples	0.74	0.57	0.79	0.77	0.72	0.98	1.06	0.85	0.82	0.67	0.81	0.95	0.84	0.75	0.79

LLOQ, lower limit of quantitation; PnPs, pneumococcal polysaccharide.

**(v) Selectivity.** The average recovery of known PnPs spike concentrations at each predilution level of urine were within 80 to 125% for 14 of the 15 STs (Table S4). The average recovery for ST 19A was 71% in neat urine and 74% in 1:2-diluted urine; however, for the remaining prediluted samples, recovery ranged from 81 to 104%. Considering that the SSUAD assay can precisely measure very low levels of ST 19A (LLOQ: 0.0078 ng/mL), the magnitude of interference was not judged to be a disqualifying characteristic.

10.1128/msphere.00114-22.4 TABLE S4 Selectivity. Average percent recovery by serotype and urine predilution level during validation. Download Table S4, DOCX file, 0.03 MB. Copyright © 2022 Rajam et al. 2022 Rajam et al. https://creativecommons.org/licenses/by/4.0/ This content is distributed under the terms of the Creative Commons Attribution 4.0 International license.

**(vi) Parallelism.** The overall dilution bias estimates per 10-fold dilution were between −12% and 28% for 14 of the 15 V114 STs and 97% for ST 1 (Fig. S1).

10.1128/msphere.00114-22.5 FIG S1 Parallelism during validation. Dilution-corrected concentrations by serotype (PnPs spiked into eight individual urine samples). Download FIG S1, TIF file, 0.9 MB. Copyright © 2022 Rajam et al. 2022 Rajam et al. https://creativecommons.org/licenses/by/4.0/ This content is distributed under the terms of the Creative Commons Attribution 4.0 International license.

**FIG 4:**
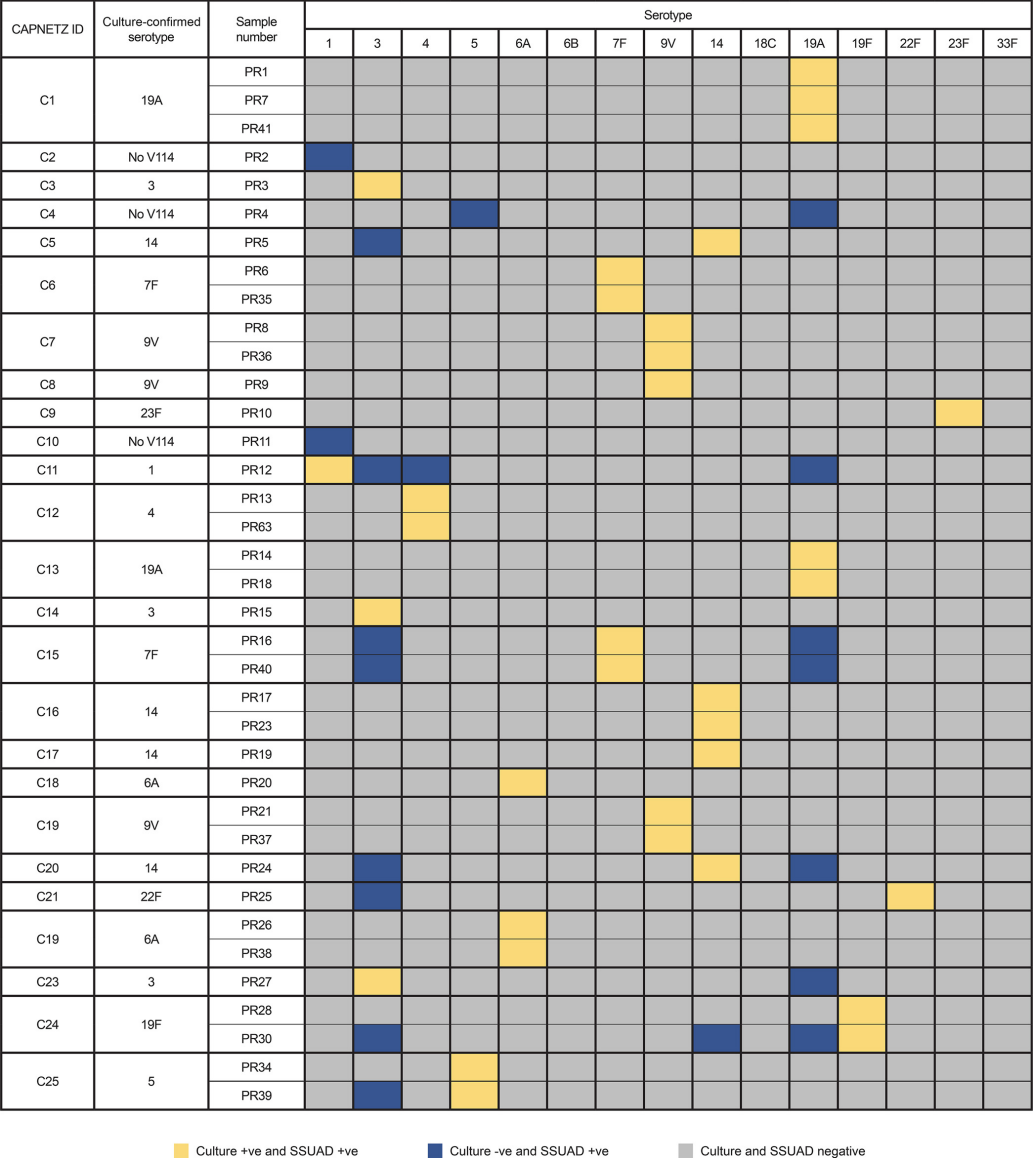
SSUAD validation with clinical samples. SSUAD, serotype-specific urine antigen detection.

**(vii) Clinical assay validation.** A positive result in the SSUAD assay was defined as a measured concentration ≥2-fold higher than the LLOQ. Quantitative results for each sample tested with the SSUAD assay were compared with the clinical culture results obtained previously and shown in Fig. 4.

A total of 129 tests were run with 34 clinical (Competence Network for Community-Acquired Pneumonia Study Group [CAPNETZ]) samples positive for V114 STs. Each sample was tested up to four times in separate runs, except for three samples without a sufficient volume. The SSUAD assay detected the appropriate culture-confirmed V114 ST in 128 of 129 instances (Fig. 4).

The levels of nonspecific background were assessed in determining the positivity cutoffs in the clinical validation study, as each of these samples was expected to test positive for a very limited number of serotypes; they were also expected to test negative for the other V114 serotypes. Albeit roughly approximated, the 2× LLOQ positivity cutoffs were selected such that the vast majority of expected positives exceeded the positivity cutoff for the anticipated serotype and the vast majority of all other serotypes had measured concentrations that were below the positivity cutoff. As noted above, for the CAPNETZ samples, the SSUAD assay detected the appropriate culture-confirmed V114 ST in 128 of 129 instances. In terms of potential false positives for the SSUAD when applying the 2× LLOQ positivity cutoffs, positive results for V114 STs other than those previously identified by positive culture were obtained for only six samples for ST 3 (range from 0.017 to 139 ng/mL) and five samples for ST 19A (range from 0.019 to 0.06 ng/mL), although most were only slightly above the 2× LLOQ cutoff of 0.016 ng/mL for ST 3 and ST 19A.

## DISCUSSION

Despite the availability of robust vaccination options, PD continues to be a major cause of respiratory morbidity and mortality. Although there is a database of ST distribution and disease burden for IPD, without sensitive assays, data for nonbacteremic PDs (NBPD) remain scarce. To address this unmet need, we developed a noninvasive urine antigen assay, the SSUAD assay, a Luminex-based multiplex assay validated to detect and quantify PnPs of 15 V114 STs in adult urine.

Early *in vitro* assays developed to detect PnPs in clinical specimens (including urine [32]) included latex agglutination, countercurrent immunoelectrophoresis, and radioimmunoassay (33–35). Later, an indirect sandwich enzyme-linked immunosorbent assay (ELISA) was developed to detect ST-specific PnPs in urine (36). These assays lacked sensitivity, required large specimen volumes, or were low throughput and/or laborious. Luminex xMAP technology enables capture of up to 100 analytes per well, equating to 100 individual ELISAs. Leveraging this high-throughput system together with ST-specific mAbs, the earliest attempt to detect PnPs in urine was a competitive inhibition assay using the pneumococcal reference serum 89SF (37). We have developed and validated a novel 15-plex SSUAD assay to detect and quantify V114 ST-specific PnPs in adult urine.

The SSUAD assay can quantify 15 V114 ST-specific PnPs in a single reaction. To ensure assay transferability to a laboratory setting, standardized protocols, reference standards, quality controls, mAbs, and customized data analysis software were developed.

The SSUAD assay is accurate, precise, highly sensitive, and remarkably specific. Some cross-reactivity was observed for ST 19F at very low concentrations (0.485 ng/mL), prompting recalibration of the LLOQ to 1.0 ng/mL for this ST. This was expected, as previous reports have shown cross-reactivity between PnPs-specific antibodies (38, 39). High specificity and sensitivity in this SSUAD assay can be attributed to the use of ST-specific mAbs for both capture and detection, a distinction from previously reported urine antigen detection assays that used polyclonal antibodies for detection (25, 40). Use of detection mAbs may also have conferred high assay sensitivity (0.007 to 1.0 ng/mL) and throughput with same-day assay capability.

Assay precision against V114 STs was confirmed during validation with clinical samples. Most importantly, the SSUAD correctly identified V114 STs in each of the culture-positive CAPNETZ clinical urine samples, demonstrating 99% (128/129) clinical specificity. This was much higher than the previously reported urine antigen detection specificity (79.3%) (40). The heightened assay sensitivity may have contributed to SSUAD positivity of some STs in clinical samples in addition to culture-positive STs. Multiple ST-specific PnPs were also reported previously with multiplex urine antigen detection (25). Conversely, the propensity to miss a serotype in a specimen with mixed STs using latex agglutination or Quellung reaction serotyping cannot be ruled out based on low sensitivity and subjectivity.

Urine is a complex matrix (41) comprising organic compounds and electrolytes. Hence, it is crucial to ensure minimal interference in this multiplex indirect PnPs capture assay. Our selectivity data demonstrated recovery of V114 ST-specific PnPs in urine from different donors with accuracy and minimal matrix interference. In addition, assay parallelism suggests that sample dilution with reproducible outcomes is possible throughout the quantifiable range of the SSUAD assay.

Assay precision to factors that are likely to vary during routine assay performance, such as bead lots, analysts, and Luminex readers, is a key characteristic that ensures transferability of SSUAD between laboratories without further optimization. SSUAD satisfies these criteria, with high levels of precision (7.1 to 14.2%) in the qualification study using spiked samples and in the validation study using clinical samples (3.2 to 24.9%). SSUAD utilizes magnetic Luminex beads instead of the polystyrene beads used previously (25, 37, 40), allowing automation of wash steps and improving assay throughput to support large surveillance or clinical studies.

### Relevance of the SSUAD assay.

S. pneumoniae causes a wide range of invasive and noninvasive PDs, with potentially fatal outcomes. Although IPD is diagnosed using blood culture, yielding ST information on the causal organism, identification of STs driving NBPD remains elusive. STs responsible for NBPD may serve as a reservoir that replaces the respiratory niche vacated by vaccine STs after effective vaccinations (4–7). A robust assay is needed to interrogate the pneumococcal ST landscape, especially in NBPD, and guide the development of next-generation vaccines. For example, the increasing prevalence of nonvaccine STs, such as 22F and 33F, may increase the incidence of non-vaccine-type PD in the future (28, 42). Hence, monitoring changes in STs associated with PD is crucial to assess the effectiveness of existing vaccines and direct future vaccine development and deployment.

Studies of pneumococcal ST replacement in CAP are scarce and hampered by the low frequency of proactive bacterial testing in clinical settings (43). The SSUAD assay described in this report will help assess the effectiveness of V114 against nonbacteremic pneumococcal pneumonia (NBPP) in ongoing vaccination programs and help monitor the changing burden of PD and the distribution of STs.

### Utility of the SSUAD assay.

Compared with traditional culture assays, the SSUAD assay has advantages. Urine samples are relatively easy to collect and can be stored frozen in batches for later analysis. In contrast, respiratory samples used for bacterial cultures can be difficult to acquire from older or sicker patients (43). Equipment and reagents required for the SSUAD assay are widely available and well established for routine laboratory purposes. Turnaround time for the SSUAD assay is relatively short, compared with the time required to conduct a culture-based Quellung or latex agglutination reaction, and the results from the SSUAD assay are quantifiable. Although a card-based assay is available that detects a common pneumococcal cell wall antigen (BinaxNOW; Abbott, CA, USA), this test does not measure specific STs present in the sample, which is vital for surveillance studies (25).

### Limitations of the SSUAD assay.

The SSUAD assay currently focuses on the 15 pneumococcal STs covered by V114. For assessments of precision, the number of clinical samples tested during the validation study was relatively low; however, the number of spiked samples tested during the qualification study was high, supporting the robustness of the results. The lower-than-expected recovery for ST 19A in assessments of relative accuracy and selectivity was the only exception to achieving the validation criteria in these experiments. However, the magnitude of potential interference due to the urine matrix was not judged to be disqualifying, given that the assay LLOQ permitted measurement of extremely low levels of ST 19A. It should also be noted that the two CAPNETZ samples with positive culture results for ST 19A were both correctly identified as ST 19A by the SSUAD assay. Finally, compared with the clinical positivity thresholds (~2× to 331× LLOQ) established for previous reported urine antigen detection assays (25), SSUAD assay analytical cutoffs were set comparatively very low (2× LLOQ). Additional nonpneumococcal clinical sample testing is planned to establish ST-specific clinical positive thresholds.

### Conclusions.

SSUAD is a multiplex assay capable of detecting and quantifying 15 V114 pneumococcal ST-specific capsular polysaccharides in adult urine. SSUAD is qualified and validated as a highly sensitive and specific assay, with acceptable accuracy and precision, for supporting surveillance studies to assess the pneumococcal ST-specific disease burden in NBPP, to assess the impact of pneumococcal vaccines on CAP, and to help inform next-generation vaccine development.

## MATERIALS AND METHODS

### Assay development and optimization.

**(i) Conjugation of monoclonal antibodies to Luminex beads.** Pairs of mAbs (Merck) recognizing each V114 ST were used as primary capture and secondary detection reagents. Human or murine ST-specific primary capture mAbs were coupled to 15 different Luminex bead regions, using manufacturer-recommended diimide conjugation chemistry (44). Briefly, 12.5 × 10^6^ Luminex beads (6.5-μm diameter) were activated in 500 μL activation buffer (pH 6.0 ± 0.05) containing 100 mM 2-[*N*-morpholino]ethanesulfonic acid hydrate (MES; Sigma, USA), 2.5 mg *N*-(3-dimethylaminopropyl)-*N*′-ethylcarbodiimide (EDC; Thermo Fisher, USA), and 2.5 mg *N*-hydroxysulfosuccinimide (NHS; Thermo Fisher, USA). The activated beads were conjugated to individual capture mAbs at different concentrations (0.125 to 2.0 μg/mL). The conjugated beads were washed and stored in 1 mL phosphate-buffered saline (PBS) with 0.05% Tween 20 (PBST; pH 7.2 ± 0.2; Thermo Fisher, USA), 1% bovine serum albumin (BSA; Sigma, USA), and 0.05% sodium azide (Thermo Fisher, USA) at 2 to 8°C in the dark. The PnPs in the sample were captured in a sandwich binding format by the ST-specific primary (human or mouse) and secondary (rabbit) mAbs. Phycoerythrin (PE)-coupled tertiary anti-rabbit reporter antibodies bind to the secondary mAbs to allow detection and quantitation of the captured PnPs using the Luminex microfluidics system (Fig. 1).

**(ii) Incubation time and conditions.** Incubation time, reagent concentration, and reaction conditions required for each assay step were optimized in singleplex format before adaptation to the multiplex format. To optimize primary, secondary, and tertiary mAb concentrations for the SSUAD assay, 15 PnPs were tested separately at three concentrations. Pneumococcal ST-specific mAbs were conjugated to Luminex beads at three concentrations and tested against three dilutions of secondary and tertiary mAbs.

**(iii) Adaptation to a multiplex assay.** The Luminex xMAP technology allows simultaneous detection of multiple STs of PnPs in a single well, reducing sample volume and assay time. The Luminex reader uses a flow cell to align the beads so that each bead can be individually interrogated for its spectral signature and PE-dependent fluorescence intensity. The test PnPs in the sample is determined by interpolating median fluorescence intensity (MFI) from the serially diluted PnPs reference curve (Fig. 1).

Optimal primary and secondary mAb concentrations determined from singleplex experiments were tested in the multiplex format, and adjustments were made for few STs. Statistical software R (45) and Design-Expert (Stat-Ease, MN) were used to optimize incubation times, assay consistency, and throughput in the multiplex format. Specificity of the PnPs mAbs used in the multiplex format was confirmed by comparing urine samples spiked with only one of each V114 ST and samples containing the other 14 of the 15 V114 STs.

**(iv) Reference standard and controls.** Commercially available urine samples from healthy adults (Biological Specialty Corp., PA) treated with PIPES [piperazine-*N*,*N*′-bis(2-ethanesulfonic acid); Sigma, USA] to a final concentration of 25 mM, prescreened with SSUAD as PnPs negative, were used as no-spike (NS) negative controls. Reference standard and QCs were prepared by spiking NS urine with specific concentrations of full-length vaccine-grade PnPs (Merck) and stored below −60°C.

To allow quantification of V114 PnPs in urine samples, a reference standard containing PnPs from all 15 V114 STs was prepared, and a standard curve was generated by diluting 2-fold for 11 dilutions with assay buffer (PBST with 0.05% casein; pH 7.2 ± 0.2), with in-well starting concentrations ranging from 0.5 to 20 ng/mL. QC samples used in each run contained high, medium, low, and no PnPs concentrations in the dynamic range of the standard curve for each ST. To process the raw MFI values, a Microsoft Excel workbook was created to perform the weighted four-parameter logistic (4PL) fitted to the standard curve, back-calculate the antigen concentrations for the QCs, and test samples from the fitted standard curve and perform the method-specified validity checks.

**(v) Optimized assay procedure.** The urine samples were treated with PIPES to a final concentration of 25 mM and stored below −60°C. The urine samples were stable at room temperature (22 to 28°C) for up to 8 h, at 2 to 8°C for up to 24 h, and up to 5 freeze–thaw cycles below −60°C. The optimized SSUAD assay was conducted at 26°C ± 2°C in 96-well plates agitated on a plate shaker (Fig. 1). Primary incubation of reference standard, QCs, and test samples (50 μL/well) with capture mAb-conjugated beads (50 μL/well) lasted 2 h ± 10 min. Subsequent incubations with secondary and tertiary antibodies (100 μL/well) were for 45 min ± 5 min each. Between incubation steps, plates were washed with PBST. Following the incubation of the tertiary antibodies, Luminex beads were resuspended in (100 μL/well) PBS (pH 7.2 ± 0.2) and analyzed using a Luminex 200 reader (BioPlex 200).

**(vi) Assay qualification.** The purpose of qualification experiments was to assess the accuracy, precision, specificity, selectivity, and parallelism of the SSUAD assay as described below. Qualification samples were stored below −60°C.

**(vii) Accuracy.** Each of eight different NS urine samples was aliquoted and spiked with 11 concentrations of the 15-PnPs reference standard (2-fold difference between spikes) with a corresponding NS control. Relative accuracy at a particular spike concentration was considered acceptable if the average recovery across the eight samples was between 80% and 125% of the known spike concentration.

PnPs recovery for each spiked test sample was assessed using the following equation: % relative accuracy = 100 × (observed concentration/expected concentration), where the observed concentration is the PnPs concentration obtained by interpolating samples against an 11-point reference standard curve tested on the same plate and the expected concentration is the concentration spiked into the sample. Observed concentrations were corrected for matrix effects by subtracting the median concentrations of the NS samples (when measurable) from the observed concentration of the spiked sample.

**(viii) Precision.** Sixteen individual NS samples were spiked with one of 16 concentrations of a reference standard containing either full-length or fragmented PnPs of the 15 V114 STs. The concentration range of spiked PnPs spanned the range of the reference standard. The set of precision samples were tested across eight runs, evenly divided between two analysts. Four different Luminex readers (Bioplex 200) were used to read assay plates. The target for acceptable precision for each V114 ST was a %CV of <25% across all replicates. Differences in concentration results among the levels of precision factors, analysts, and Luminex reader were considered acceptable if the average difference in test sample concentration was <30%.

Intra-assay precision was assessed using data from the PnPs-spiked samples that were tested twice within each assay run. Total or intermediate assay precision was assessed for each individual test sample using all determinable concentrations. Geometric mean concentration (GMC) and %CV were calculated separately for each test sample by variance component analysis using the Mixed Procedure in SAS. The mixed model contained random terms for *Analyst* and *Run within Analyst*. Variance component analysis was also used to assess total assay precision across the set of test samples in which the GMC was within the determined LOQ. The mixed model to assess total assay precision across test samples contained a fixed term for *Sample* and random terms for *Analyst*, *Run within Analyst*, and their interactions with *Sample*. Total or intermediate precision was calculated as $100\%\times \sqrt{e^{V}-1}\;$ where *V* denotes the sum of the variance component estimates. Estimates of the differences among the levels within each precision factor were the differences between the least-squares means obtained by fitting a model containing fixed terms for *Sample*, *Analyst*, and *Luminex Instrument* to the natural log-transformed PnPs concentrations.

**(ix) Sensitivity and LOQ.** The LOQ were the lowest and highest concentrations of PnPs detected with relative accuracy within 80 to 125% of the known spike concentration and with total assay variability of <25%. The LOQ were determined by spiking the PnPs reference standard at 11 concentrations (2-fold difference between spikes) into aliquots of eight different NS urine samples. The LOQ were also restricted to be within the concentrations of the second and tenth points of the standard curve.

**(x) Specificity.** Analytical specificity of the SSUAD assay was determined by assessing its ability to measure and report the presence of specific V114 ST PnPs in the sample. In each of the two runs, 15 NS samples were spiked with 14 of the 15 V114 ST PnPs, with each sample missing a different ST. The spiked concentration of each PnPs corresponded to the fourth highest quantifiable concentration of the standard curve; a level sufficiently high to detect and quantify cross-reactivity among STs, if present. An NS control and a positive control consisting of pooled urine containing all 15 PnPs were also included in each run.

The GMCs across the two runs were reported for each V114 ST spiked in each urine sample.

**(xi) Selectivity.** A panel of eight different NS urine samples were diluted 2-fold with increasing amounts of assay buffer (0.05% casein in PBST), creating a series of eight dilutions ranging from neat to 1:128. Selectivity of the SSUAD assay was evaluated by measuring a known concentration of PnPs reference standards spiked into each diluted urine sample. The final in-well concentration of PnPs corresponded to the fifth highest concentration of the standard curve, chosen to allow quantitation at higher levels of response if the urine matrix depressed the measured concentration.

Mean percentage recovery and 95% confidence intervals were calculated for each level of diluted urine for each V114 ST.

**(xii) Parallelism.** The parallelism of the SSUAD assay was assessed by spiking the PnPs reference standard into each of eight different NS samples and then serially diluting each spiked sample 4-fold in assay buffer for a four-point dilution series (neat, 1:4, 1:16, and 1:64). Each sample contained a final concentration of spiked PnPs corresponding to the third-highest concentration of the standard curve. This concentration was chosen to ensure that the neat sample and each of the subsequent dilutions were quantifiable. The predetermined acceptance criterion for parallelism was that the dilution bias per 10-fold dilution be less than 2-fold.

Parallelism was estimated using a mixed model that included fixed terms for samples and the average dilution effect (slope), along with a random term representing the variability in the dilution effect across the individual test samples. Dilution bias per 10-fold dilution was calculated as 100 × (10*^b^* – 1), where *b* represents the estimate of the average dilution effect (slope) from the mixed model.

### Clinical validation: conditions unique to validation experiments.

Validation experiments were conducted to ensure that the SSUAD assay performance characteristics were consistent with the acceptance criteria established during assay qualification using clinical samples. Validation samples were stored below −60°C.

Clinical urine samples from 25 patients with CAP, confirmed by positive culture from blood, pleural fluid, or other sterile sites, were acquired from CAPNETZ, an ongoing prospective survey of CAP in Germany (23, 43). Ten of the 25 CAPNETZ samples were divided into two aliquots, while one other patient sample was divided into three aliquots so that, along with the remaining 14 samples, the study panel included 37 individual clinical CAP samples.

Clinical urine samples from 16 patients with NBPP and confirmed vaccine ST positivity collected in the CORE PNEUMO US surveillance study (46) were also included in the validation experiments. Three of the 16 NBPP patient samples were divided into three aliquots, two other samples were divided into two aliquots, and one sample was divided into four aliquots so that, along with the remaining 10, the study panel included 27 individual clinical NBPP samples.

For precision, the 37 CAPNETZ samples and the 27 CORE samples were tested four times each at a dilution factor determined during prescreening before assay validation. A total of eight one-plate runs were performed, with half of the 64 samples tested within each run. The eight runs were evenly divided between two analysts, with ≥1 week between each analyst’s first two and last two runs. For the assessment of assay precision, the 64 urine samples were treated as independent samples in the analysis, and, therefore, only total assay precision was estimated.

### Data availability.

Data will be made available upon request after product approval in the United States and EU or after product development is discontinued. Merck Sharp & Dohme LLC, a subsidiary of Merck & Co., Inc., Rahway NJ, USA’s data sharing policy, including restrictions, is available at http://engagezone.msd.com/ds_documentation.php.
