# Supplementary material for: Development and Validation of a Sensitive and Robust Multiplex Antigen Capture Assay to Quantify Streptococcus pneumoniae Serotype-Specific Capsular Polysaccharides in Urine
Source: mSphere. 2022 Aug 1;7(4):e00114-22. doi: 10.1128/msphere.00114-22 (PMC9429912; doi:10.1128/msphere.00114-22)
Supplement: TABLE S1 [file msphere.00114-22-s0001.docx]

**Table S1** Control sample acceptance limits (ng/mL) by control sample and serotype

| Serotype | C1 | C2 | C3 | C4 |
| --- | --- | --- | --- | --- |
| 1 | (0.275–0.735) | (0.085–0.227) | (0.788– >ULOQ) | (N/A–<LLOQ) |
| 3 | (0.292–0.783) | (0.074–0.197) | (0.012–0.033) | (N/A–<LLOQ) |
| 4 | (1.446–3.87) | (0.339–0.906) | (0.081–0.218) | (N/A–<LLOQ) |
| 5 | (1.296–3.468) | (0.308–0.824) | (0.075–0.2) | (N/A–<LLOQ) |
| 6A | (1.606–4.299) | (0.382–1.022) | (0.087–0.233) | (N/A–<LLOQ) |
| 6B | (0.335–0.897) | (0.081–0.217) | (0.018–0.049) | (N/A–<LLOQ) |
| 7F | (6.778–18.144) | (1.638–4.504) | (0.399–1.069) | (N/A–<LLOQ) |
| 9V | (0.51–1.365) | (0.123–0.329) | (0.027–0.071) | (N/A–<LLOQ) |
| 14 | (1.203–3.22) | (0.286–0.766) | (0.07–0.189) | (N/A–<LLOQ) |
| 18C | (1.314–3.516) | (0.337–0.902) | (N/A–<LLOQ) | (N/A–<LLOQ) |
| 19A | (0.318–0.85) | (0.071–0.189) | (0.015–0.04) | (N/A–<LLOQ) |
| 19F | (5.475–14.656) | (1.369–3.665) | (N/A–<LLOQ) | (N/A–<LLOQ) |
| 22F | (1.106–2.961) | (0.319–0.853) | (N/A–<LLOQ) | (N/A–<LLOQ) |
| 23F | (13.672–36.599) | (3.285–8.795) | (0.826–2.212) | (N/A–<LLOQ) |
| 33F | (1.259–3.37) | (0.31–0.83) | (N/A–0.138) | (N/A–<LLOQ) |
