# Supplementary material for: Development and Validation of a Sensitive and Robust Multiplex Antigen Capture Assay to Quantify Streptococcus pneumoniae Serotype-Specific Capsular Polysaccharides in Urine
Source: mSphere. 2022 Aug 1;7(4):e00114-22. doi: 10.1128/msphere.00114-22 (PMC9429912; doi:10.1128/msphere.00114-22)
Supplement: TABLE S2 [file msphere.00114-22-s0002.docx]

**Table S2** SSUAD assay validation parameters **(1, 2)**

| **Parameter** | **Definition** | **Acceptance criterion for each serotype** |
| --- | --- | --- |
| Limits of quantitation (lower and upper limits of quantitation) | An assay's quantifiable range is defined by its LLOQ and ULOQ. The LLOQ is the lowest amount of an analyte that can be quantitatively determined with acceptable precision and accuracy. The ULOQ is the highest amount of an analyte in a sample that can be quantitatively determined with acceptable precision and accuracy. | The LLOQ must be ≤1 ng/mL and the quantifiable range must be ≥10-fold. The LLOQ is restricted to be ≥LOD. |
| Total precision | Precision is the closeness of agreement (ie, degree of scatter) among a series of measurements obtained from multiple sampling of the same homogenous sample under the prescribed conditions. | Total assay variability should be less than 25% CV and differences in test sample concentrations between the precision factor levels should be <30%. |
| Accuracy | Accuracy is the degree of closeness of the determined value to the nominal or known true value under prescribed conditions. | The % relative accuracy estimates should be between 80% and 125% throughout the limits of quantitation. |
| Specificity | Specificity is the ability of the method to assess, unequivocally, the analyte in the presence of other components that are expected to be present in the sample. | The average measured concentration for the spiked PnPs will be within 2-fold its spike concentration, and that the absent PnPs will generate a result that is below its determined LLOQ. |
| Selectivity | Selectivity is the extent to which the method can determine a particular compound in the analyzed matrices without interference from matrix components. | The average % recovery should be between 80% and 125% at each pre-dilution of the urine matrix. |
| Parallelism | Parallelism is defined as a parallel relationship between the calibration curve and serially diluted study samples to detect any influence of dilution on analyte measurement. | The dilution-bias per 10-fold dilution should be less than two-fold throughout the evaluated dilution range. |

1. European Medicines Agency. 14 March 2019 2019. ICH M10 on bioanalytical method validation. <https://www.ema.europa.eu/en/ich-m10-bioanalytical-method-validation>. Accessed 10 May, 2022.

2. U.S. Food and Drug Administration. 29 April 2020 2018. Bioanalytical Method Validation Guidance for Industry. <https://www.fda.gov/regulatory-information/search-fda-guidance-documents/bioanalytical-method-validation-guidance-industry>. Accessed 10 May, 2022.
