# Supplementary material for: Development and Validation of a Sensitive and Robust Multiplex Antigen Capture Assay to Quantify Streptococcus pneumoniae Serotype-Specific Capsular Polysaccharides in Urine
Source: mSphere. 2022 Aug 1;7(4):e00114-22. doi: 10.1128/msphere.00114-22 (PMC9429912; doi:10.1128/msphere.00114-22)
Supplement: TABLE S3 [file msphere.00114-22-s0003.docx]

**Table S3** Summary of control failures by serotype and control sample during validation

| **Type** | **Number of total tested plates (Number of failures)** | | | | |
| --- | --- | --- | --- | --- | --- |
|  | **C1** | **C2** | **C3** | **C4** | **Plate (C1–C4)** |
| 1 | 36 (1) | 36 (0) | 36 (1) | 36 (0) | 36 (0) |
| 3 | 36 (1) | 36 (0) | 36 (0) | 36 (0) | 36 (0) |
| 4 | 36 (1) | 36 (0) | 36 (1) | 36 (0) | 36 (0) |
| 5 | 36 (1) | 36 (0) | 36 (0) | 36 (0) | 36 (0) |
| 6A | 36 (1) | 36 (0) | 36 (0) | 36 (0) | 36 (0) |
| 6B | 36 (0) | 36 (0) | 36 (0) | 36 (0) | 36 (0) |
| 7F | 36 (1) | 36 (0) | 36 (0) | 36 (0) | 36 (0) |
| 9V | 36 (1) | 36 (0) | 36 (2) | 36 (0) | 36 (0) |
| 14 | 36 (1) | 36 (0) | 36 (1) | 36 (0) | 36 (0) |
| 18C | 36 (2) | 36 (1) | 36 (0) | 36 (0) | 36 (0) |
| 19A | 36 (1) | 36 (0) | 36 (3) | 36 (0) | 36 (0) |
| 19F | 36 (2) | 36 (0) | 36 (0) | 36 (0) | 36 (0) |
| 22F | 36 (1) | 36 (0) | 36 (0) | 36 (0) | 36 (0) |
| 23F | 36 (2) | 36 (0) | 36 (3) | 36 (0) | 36 (1) |
| 33F | 36 (2) | 36 (0) | 36 (3) | 36 (0) | 36 (0) |
