# Supplementary material for: Development and Validation of a Sensitive and Robust Multiplex Antigen Capture Assay to Quantify Streptococcus pneumoniae Serotype-Specific Capsular Polysaccharides in Urine
Source: mSphere. 2022 Aug 1;7(4):e00114-22. doi: 10.1128/msphere.00114-22 (PMC9429912; doi:10.1128/msphere.00114-22)
Supplement: TABLE S4 [file msphere.00114-22-s0004.docx]

**Table S4** Selectivity: Average percent recovery by serotype and urine pre-dilution level during validation

| **Type** | **Pre-dilution** |  | **Recovery (%)** | |
| --- | --- | --- | --- | --- |
|  |  | **N** | **Average** | **95% CI** |
| 1 | 1 | 8 | 99.0 | 82.5–118.2 |
|  | 2 | 8 | 101.0 | 91.2–112.4 |
|  | 4 | 8 | 106.0 | 98.8–113.9 |
|  | 8 | 8 | 109.0 | 101.7–117.7 |
|  | 16 | 8 | 111.0 | 99.1–123.3 |
|  | 32 | 8 | 108.0 | 101.0–115.4 |
|  | 64 | 8 | 110.0 | 100.1–120.8 |
|  | 128 | 8 | 112.0 | 101.5–122.6 |
| 3 | 1 | 8 | 100.0 | 93.3–108.2 |
|  | 2 | 8 | 100.0 | 95.1–105.8 |
|  | 4 | 8 | 104.0 | 96.7–111.3 |
|  | 8 | 8 | 104.0 | 96.6–111.1 |
|  | 16 | 8 | 104.0 | 94.9–113.1 |
|  | 32 | 8 | 102.0 | 92.0–112.9 |
|  | 64 | 8 | 104.0 | 95.0–113.4 |
|  | 128 | 8 | 106.0 | 97.2–115.5 |
| 4 | 1 | 8 | 92.0 | 79.6–105.6 |
|  | 2 | 8 | 91.0 | 79.7–103.6 |
|  | 4 | 8 | 96.0 | 86.7–105.5 |
|  | 8 | 8 | 100.0 | 91.2–109.9 |
|  | 16 | 8 | 103.0 | 93.8–112.4 |
|  | 32 | 8 | 100.0 | 92.6–108.7 |
|  | 64 | 8 | 100.0 | 92.0–109.1 |
|  | 128 | 8 | 108.0 | 98.9–117.0 |
| 5 | 1 | 8 | 105.0 | 96.7–113.7 |
|  | 2 | 8 | 107.0 | 100.1–114.0 |
|  | 4 | 8 | 105.0 | 97.1–114.4 |
|  | 8 | 8 | 108.0 | 102.2–113.9 |
|  | 16 | 8 | 105.0 | 97.4–113.1 |
|  | 32 | 8 | 107.0 | 100.6–113.9 |
|  | 64 | 8 | 107.0 | 99.0–114.7 |
|  | 128 | 8 | 113.0 | 101.3–125.0 |
| 6A | 1 | 8 | 104.0 | 91.2–118.7 |
|  | 2 | 8 | 100.0 | 93.1–107.0 |
|  | 4 | 8 | 104.0 | 93.8–114.6 |
|  | 8 | 8 | 108.0 | 101.1–114.5 |
|  | 16 | 8 | 106.0 | 98.3–113.7 |
|  | 32 | 8 | 106.0 | 98.9–113.7 |
|  | 64 | 8 | 108.0 | 100.9–115.7 |
|  | 128 | 8 | 110.0 | 103.4–117.3 |
| 6B | 1 | 8 | 101.0 | 95.0–108.1 |
|  | 2 | 8 | 98.0 | 91.7–105.7 |
|  | 4 | 8 | 103.0 | 96.4–109.1 |
|  | 8 | 8 | 100.0 | 93.7–106.2 |
|  | 16 | 8 | 101.0 | 93.0–109.0 |
|  | 32 | 8 | 100.0 | 94.0–106.5 |
|  | 64 | 8 | 102.0 | 97.7–107.4 |
|  | 128 | 8 | 105.0 | 99.8–111.0 |
| 7F | 1 | 8 | 102.0 | 91.7–112.6 |
|  | 2 | 8 | 95.0 | 86.2–103.8 |
|  | 4 | 8 | 95.0 | 89.1–101.9 |
|  | 8 | 8 | 96.0 | 90.4–102.5 |
|  | 16 | 8 | 95.0 | 86.8–104.5 |
|  | 32 | 8 | 96.0 | 89.0–104.6 |
|  | 64 | 8 | 97.0 | 89.2–105.4 |
|  | 128 | 8 | 98.0 | 91.0–105.4 |
| 9V | 1 | 8 | 96.0 | 89.5–102.9 |
|  | 2 | 8 | 99.0 | 95.4–103.7 |
|  | 4 | 8 | 102.0 | 96.1–108.0 |
|  | 8 | 8 | 103.0 | 99.5–106.9 |
|  | 16 | 8 | 102.0 | 98.0–106.7 |
|  | 32 | 8 | 102.0 | 98.1–106.9 |
|  | 64 | 8 | 106.0 | 102.4–109.4 |
|  | 128 | 8 | 107.0 | 103.7–111.1 |
| 14 | 1 | 8 | 102.0 | 91.8–112.6 |
|  | 2 | 8 | 99.0 | 91.2–108.1 |
|  | 4 | 8 | 102.0 | 90.2–115.4 |
|  | 8 | 8 | 103.0 | 94.4–112.3 |
|  | 16 | 8 | 104.0 | 94.6–115.1 |
|  | 32 | 8 | 102.0 | 92.2–111.9 |
|  | 64 | 8 | 104.0 | 95.0–114.3 |
|  | 128 | 8 | 110.0 | 99.0–121.3 |
| 18C | 1 | 8 | 91.0 | 83.5–99.0 |
|  | 2 | 8 | 90.0 | 82.8–98.5 |
|  | 4 | 8 | 90.0 | 84.7–95.4 |
|  | 8 | 8 | 95.0 | 86.8–105.0 |
|  | 16 | 8 | 99.0 | 93.4–104.0 |
|  | 32 | 8 | 91.0 | 79.8–104.3 |
|  | 64 | 8 | 91.0 | 78.4–106.5 |
|  | 128 | 8 | 101.0 | 87.5–117.2 |
| 19A | 1 | 8 | 71.0 | 58.4–86.3 |
|  | 2 | 8 | 74.0 | 61.8–88.3 |
|  | 4 | 8 | 81.0 | 69.0–95.9 |
|  | 8 | 8 | 92.0 | 81.5–104.1 |
|  | 16 | 8 | 93.0 | 80.4–106.8 |
|  | 32 | 8 | 94.0 | 82.7–107.9 |
|  | 64 | 8 | 98.0 | 87.1–109.6 |
|  | 128 | 8 | 104.0 | 91.4–119.3 |
| 19F | 1 | 8 | 104.0 | 88.7–122.6 |
|  | 2 | 8 | 103.0 | 90.5–118.2 |
|  | 4 | 8 | 102.0 | 91.3–114.3 |
|  | 8 | 8 | 105.0 | 94.7–116.2 |
|  | 16 | 8 | 105.0 | 93.1–117.6 |
|  | 32 | 8 | 103.0 | 94.3–113.0 |
|  | 64 | 8 | 107.0 | 97.2–116.7 |
|  | 128 | 8 | 114.0 | 100.9–128.8 |
| 22F | 1 | 8 | 104.0 | 94.5–113.6 |
|  | 2 | 8 | 103.0 | 95.0–111.4 |
|  | 4 | 8 | 104.0 | 97.3–112.0 |
|  | 8 | 8 | 104.0 | 96.2–111.9 |
|  | 16 | 8 | 103.0 | 93.8–113.5 |
|  | 32 | 8 | 103.0 | 95.3–111.2 |
|  | 64 | 8 | 104.0 | 98.6–110.6 |
|  | 128 | 8 | 107.0 | 97.2–118.8 |
| 23F | 1 | 8 | 96.0 | 89.6–102.1 |
|  | 2 | 8 | 93.0 | 88.3–97.3 |
|  | 4 | 8 | 93.0 | 86.3–100.2 |
|  | 8 | 8 | 94.0 | 90.4–97.2 |
|  | 16 | 8 | 94.0 | 89.1–99.7 |
|  | 32 | 8 | 93.0 | 88.6–97.8 |
|  | 64 | 8 | 97.0 | 90.7–104.0 |
|  | 128 | 8 | 100.0 | 94.4–106.7 |
| 33F | 1 | 8 | 108.0 | 101.4–115.8 |
|  | 2 | 8 | 110.0 | 104.1–115.9 |
|  | 4 | 8 | 111.0 | 106.2–117.0 |
|  | 8 | 8 | 113.0 | 108.4–118.3 |
|  | 16 | 8 | 111.0 | 105.4–117.8 |
|  | 32 | 8 | 110.0 | 104.0–117.1 |
|  | 64 | 8 | 112.0 | 105.7–118.0 |
|  | 128 | 8 | 116.0 | 108.0–124.8 |
