# Supplementary figures and images for: Development and Validation of a Sensitive and Robust Multiplex Antigen Capture Assay to Quantify Streptococcus pneumoniae Serotype-Specific Capsular Polysaccharides in Urine
Source: mSphere. 2022 Aug 1;7(4):e00114-22. doi: 10.1128/msphere.00114-22 (PMC9429912; doi:10.1128/msphere.00114-22)

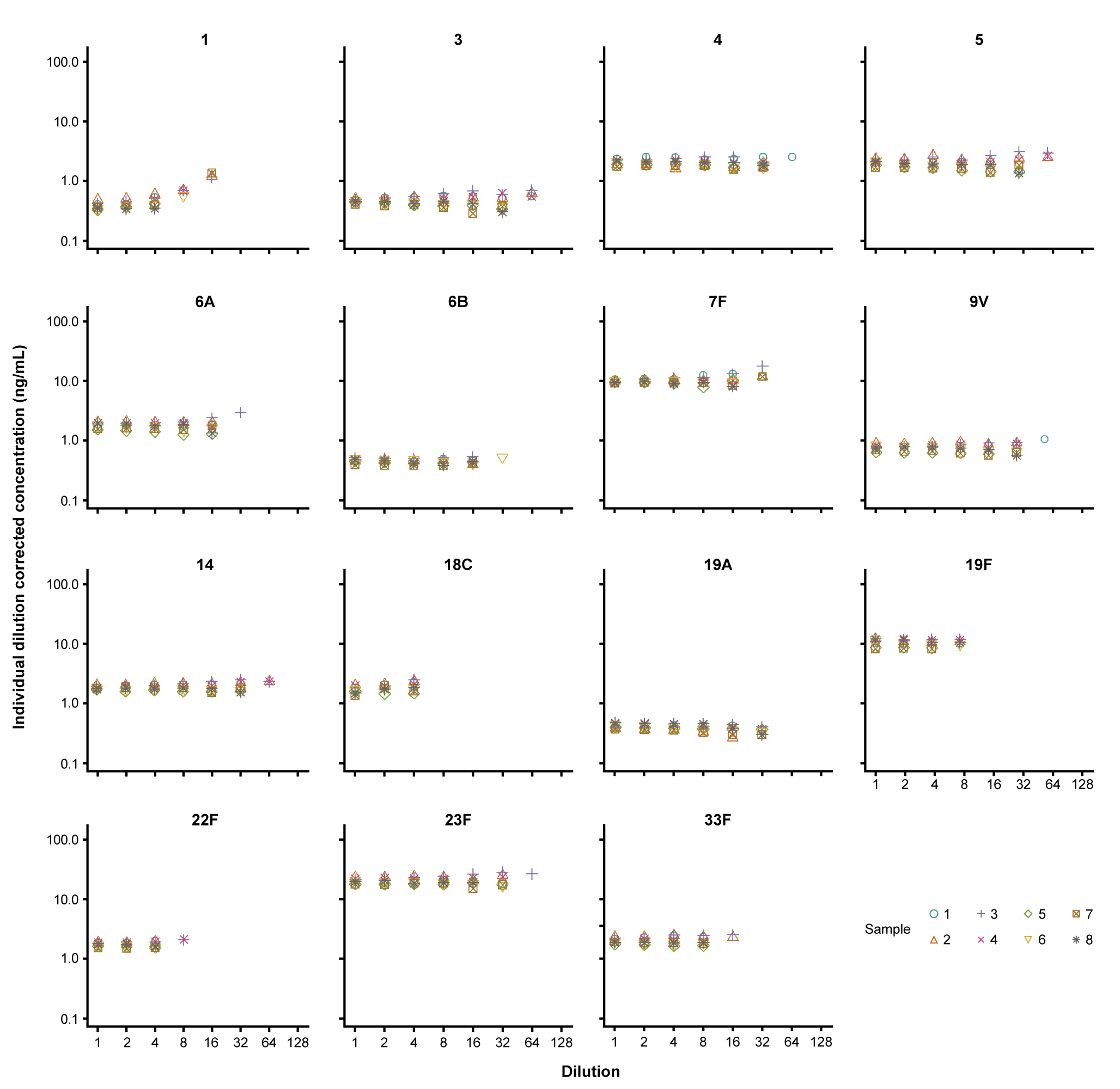

Supplement: FIG S1 [file msphere.00114-22-s0005.tif]
